# Efficient expression of an alkaline pectin lyase from *Bacillus licheniformis* in *Pichia pastoris*

**DOI:** 10.1186/s40643-024-00752-w

**Published:** 2024-04-15

**Authors:** Junyi Li, Manli Yang, Fengguang Zhao, Yaping Zhang, Shuangyan Han

**Affiliations:** 1 Guangdong Key Laboratory of Fermentation and Enzyme Engineering, School of Biology and Biological Engineering, South China University of Technology, Guangzhou, 510006 China; 2 School of Light Industry and Engineering, South China University of Technology, Guangzhou, 510006 China

**Keywords:** Alkaline pectin lyase, Efficient expression, *Pichia pastoris*

## Abstract

Pectin lyase (PMGL) is an industrially important enzyme with widespread applications in the food, paper, and textile industries, owing to its capacity for direct degradation of highly esterified pectin. In this study, PMGL-Ba derived from *Bacillus licheniformis* underwent mining and heterologous expression in *P. pastoris.* Furthermore, diverse strategies, encompassing the optimization of expression cassette components, elevation of gene dosage, and co-expression of chaperone factors, were employed to augment PMGL-Ba production in *P. pastoris*. The signaling peptide OST1-pre-α-MF-pro and promoter AOX1 were finally selected as expression elements. By overexpressing the transcription factor Hac1p in conjunction with a two-copy PMGL-Ba setup, a strain yielding high PMGL-Ba production was achieved. In shake flask fermentation lasting 144 h, the total protein concentration reached 1.81 g/L, and the enzyme activity reached 1821.36 U/mL. For further scale up production, high-density fermentation transpired in a 5 L fermenter for 72 h. Remarkably, the total protein concentration increased to 12.49 g/L, and the enzyme activity reached an impressive 12668.12 U/mL. The successful heterologous and efficient expression of PMGL-Ba not only furnishes a valuable biological enzyme for industrial applications but also contributes to cost reduction in the utilization of biological enzymes in industrial applications.

**Supplementary Information:**

The online version contains supplementary material available at 10.1186/s40643-024-00752-w.

## Introduction

Pectin, a structural polysaccharide ubiquitous in plant cell walls, consists of D-galacturonosyl residues linked by α-1,4-glycosidic bonds, imparting both rigidity and flexibility. This polymer serves as a protective agent for plants (Kaur et al. 2023; Zheng et al. 2021). However, in specific industrial scenarios, altering pectin’s structure becomes imperative to curtail energy consumption or enhance product quality. Traditional mechanical and chemical methods pose environmental risks and may harm raw materials. Enzymatic treatments emerge as preferred alternatives due to their efficiency, specificity, and environmental friendliness (Haile et al. 2022; Kaur et al. 2023). The degradation of pectin involves three primary types of pectinases: methyl deesterases (pectin methyl esterases), hydrolases (polygalacturonases), and lyases (pectin lyase) (Suberkropp and Keller 2020). Pectin methyl esterase initiates methoxylation, completing deesterification and producing pectin and methanol. Subsequently, pectin lyase and polygalacturonases preferentially act on highly esterified pectin, catalyzing hydrolytic cleavage of the α-1,4-glycosidic bond in the pectin backbone without methanol production, resulting in 6-methyl-D-galacturonic acid. This crucial step in pectin degradation, involving cutting the pectin skeleton and progressively breaking down the substance, makes pectin lyase vital for industrial environmental protection (Tasgin et al. 2020). In addition, pectin lyase consists of two lyases, polymethylgalacturonate lyase (PMGL) and polygalacturonate lyase (PGL). Currently, there are few reports of PMGL. PMGL can directly degrade highly esterified pectin, but PGL can only degrade demethylated pectic acid, and Ca^2+^ must be involved (Haile et al. 2022; Zheng et al. 2021).

Pectinase pretreatment finds diverse applications, reducing fruit juice viscosity in food processing (Rahman et al. 2020), enhancing cotton fabric absorbency in the textile industry (Aggarwal et al. 2020), and improving paper quality while decreasing chemical usage in the paper industry (Ahlawat et al. 2007; Nawawi et al. 2021; Singh et al. 2020). Notably, treating mechanical pulp with pectinase degrades pectin on fibers, reducing the amount of extractives covering the fiber surface. This weakens the bond between lignin and cellulose, refining the pulp further. The enzyme pretreatment exposes more microfibers, facilitating inter-fiber bonding and improving fiber strength, demonstrating the versatility and significance of pectinase in various industrial processes (Ahlawat et al. 2007; Li et al. 2010; Chen et al. 2020).

However, the environments in which biological enzymes are used in industrial applications are not benign, and there are stringent requirements for the characteristics of the enzymes in different industrial application environments. The environments used in the paper industry are generally alkaline and medium-high temperature environments, and digging for more alkaline pectin cleaving enzymes has become an important task, laying a solid foundation for the wide application of enzymes for the paper industry.

While the application of biological enzymes in industry is highly effective for green manufacturing, elevated production costs present a significant challenge. Hence, improving the production efficiency of enzymes and exploring high-expression strategies are crucial long-term research objectives. *P.pastoris*, with advantages such as high cell culture density, genetic manipulation, and post-translational modification of proteins, stands as a widely used bacterium for producing exogenous proteins (Ahmad et al. 2014; Vijayakumar and Venkataraman 2023). Leveraging the secretion mechanism of exogenous proteins in *P.pastoris*, extensive research has focused on optimizing strategies to enhance protein secretion. These strategies encompass the construction of expression platforms, pathway engineering modification, and optimization of culture conditions, resulting in the efficient expression of diverse exogenous proteins (Liu et al. 2022; Raschmanová et al. 2021). Specifically, within the expression system, the emphasis lies on targeting promoter strength, selecting secretion signal peptides, and optimizing codons for the target proteins in the expression cassette (Xiao et al. 2023; Xu et al. 2023). Pathway engineering modification primarily involve using relevant cofactors in the co-expression pathway to promote transcription, translation, processing and secretion of the protein, while averting stress reactions and protein degradation within the cell (Jiang et al. 2023; Zahrl et al. 2022, 2023). In the realm of culture conditions, the focus is on optimizing medium composition, pH, temperature, and implementing high-density fermentation (Dixit et al. 2022). Additionally, increasing the gene dose through in vitro constructs and in vivo screening is a commonly employed strategy to promote high expression (Peng et al. 2019; Chen et al. 2022). Although these strategies have shown success in increasing the yield of exogenous proteins, their effects can be unpredictable for different proteins and require customization. Among the studies that have been reported, Chen et al. increased the expression of alkaline pectinase (PGL) in *P. pastoris* by co-expressing molecular chaperones. The fermentation was carried out in a 3 L fermenter, and the enzyme activity of PGL reached 1362.31 U/mL after 96 h of induced fermentation (Chen et al. 2016). Yu et al. increased the alkaline pectinase activity of *Bacillus subtilis FS105* by genomic reorganization, and obtained the fusion strain *B. subtilis FS105* with the highest enzyme activity of 499 U/ml, which was 1.6-fold higher than the starting strain (Yu et al. 2019). Peng et al. constructed a recombinant pectin lyase multicopy *P. pastoris* for high-density fermentation in a 14 L fermenter, and the pectin lyase enzyme activity reached 3620 U/mg (Peng 2020). Chen et al. increased the expression of alkaline pectinase (PELA) in by two strategies: signal peptide optimization and molecular chaperone co-expression, and the enzyme activity reached 2,301.05 U/mL by high-density fermentation using a 5 L fermenter under optimal induction conditions (Chen et al. 2022). We can see that the strategies used to increase alkaline pectinase expression in these studies were limited to one or two. So there is still substantial room for improvement in optimizing the expression of pectin lyase in *P. pastoris*. It is expected that the expression of alkaline pectin lyase in *P. pastoris* in this study exceeds that of previous studies through the superposition of multiple strategies at the same level of fermentation.

In this paper, an unreported PMGL gene derived from *Bacillus licheniformis* was selected by homologous sequence comparison and heterologously expressed in *P. pastoris*. Subsequently, a strain of *P. pastoris* with high expression of PMGL-Ba was constructed by several strategies, including codon optimization, optimization of gene expression elements, increasing gene dosage and overexpression of cofactors. The objective is to provide an industrial enzyme and reduce the associated costs, addressing a critical aspect in the practical application of these enzymes in various industrial processes.

## Results

### Gene mining and sequence analysis of PMGL-Ba

**Fig. 1:**
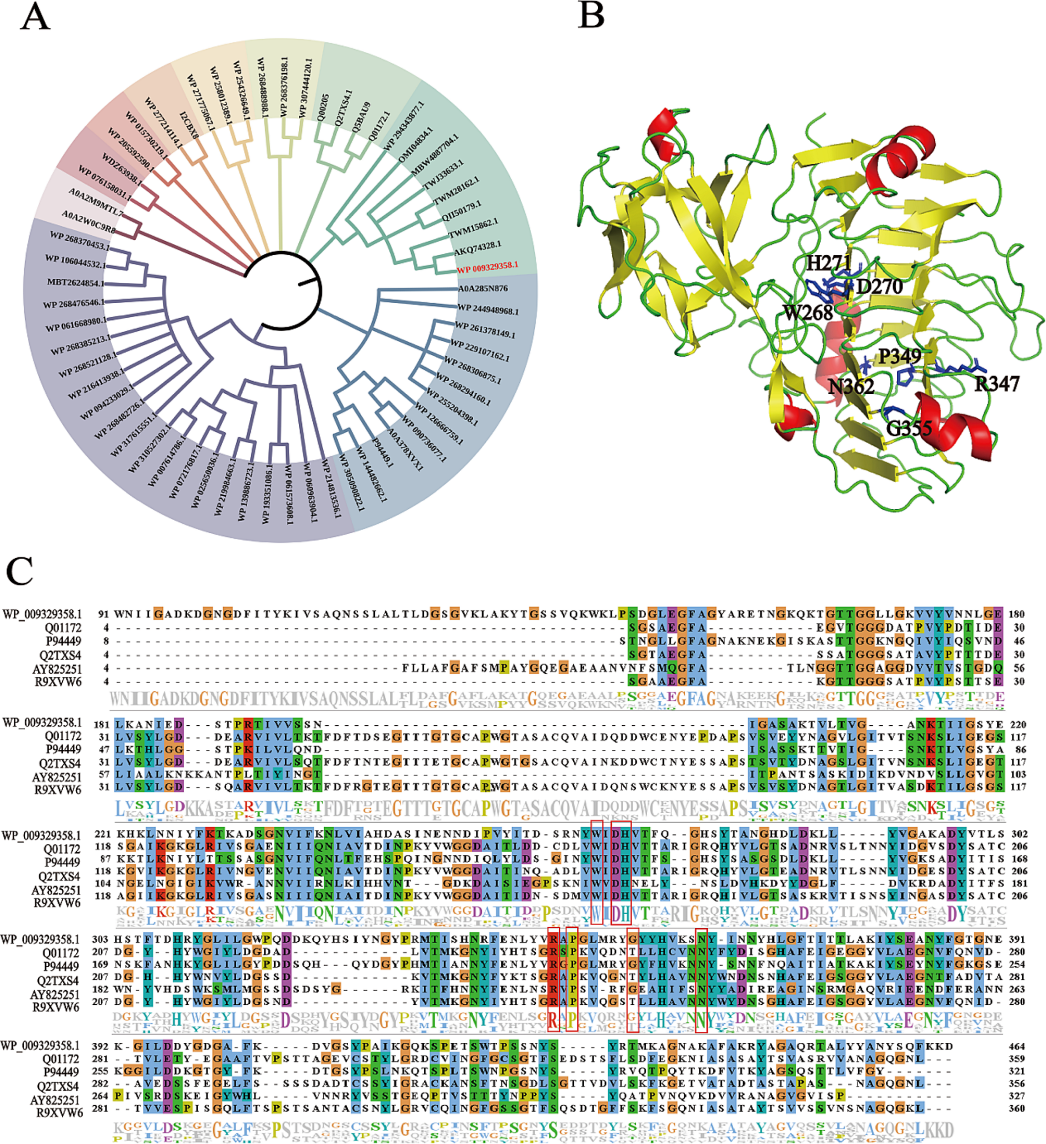
Phylogenetic analysis of PMGL-Ba. (**A**) Selection of signal peptides. (**B**) The predicted structure of PMGL-Ba. Red represents α-helix, gold represents β-sheet, and green represents random coil structure. (**C**) Sequence comparison analysis

The gene mining process for PMGL involved using a high enzyme activity pectin lyase gene as the probe sequence, with key residues serving as the screening conditions. The selection criteria included assessing sequence similarity to the probe template, predicting soluble expression of the protein, and considering the type of microorganisms of origin. Ultimately, the identified putative PMGL-Ba (NCBI Reference Sequence: WP_009329358.1) derived from *Bacillus licheniformis* was finally selected. The protein consists of 494 amino acids with a relative molecular mass of 54693.2. PMGL-Ba was found to be in the same branch as two *Bacillus*-derived pectin lyases (GenBank: OMI04834.1, QII50179.1) annotated in NCBI, and both are in the polysaccharide lyase 1 family, confirming the reliability of the results(Fig. 1A). Analysis of the protein structure diagram revealed that PMGL-Ba possesses the typical β-helical structure of pectin lyase proteins, consisting of three parallel β-strands connected through three corners (Zheng et al. 2021) (Fig. 1B). Sequence comparisons indicated that certain sequences of PMGL-Ba overlap with conserved amino acid sequences of the pectin lyase reference sequence or contain key amino acids of the catalytic activity center, suggesting potential functional similarities (Fig. 1C). In this paper, Enzyme Miner was used in conjunction with NCBI to find a functionally similar enzyme gene, PMGL-Ba. This method can find the target enzyme more quickly and reduces the cost of trial and error of wet experiments to a certain extent, and this experiment provides a good example for Enzyme Miner to mine functional enzymes.

### Biochemical characterization of PMGL-Ba

**Fig. 2:**
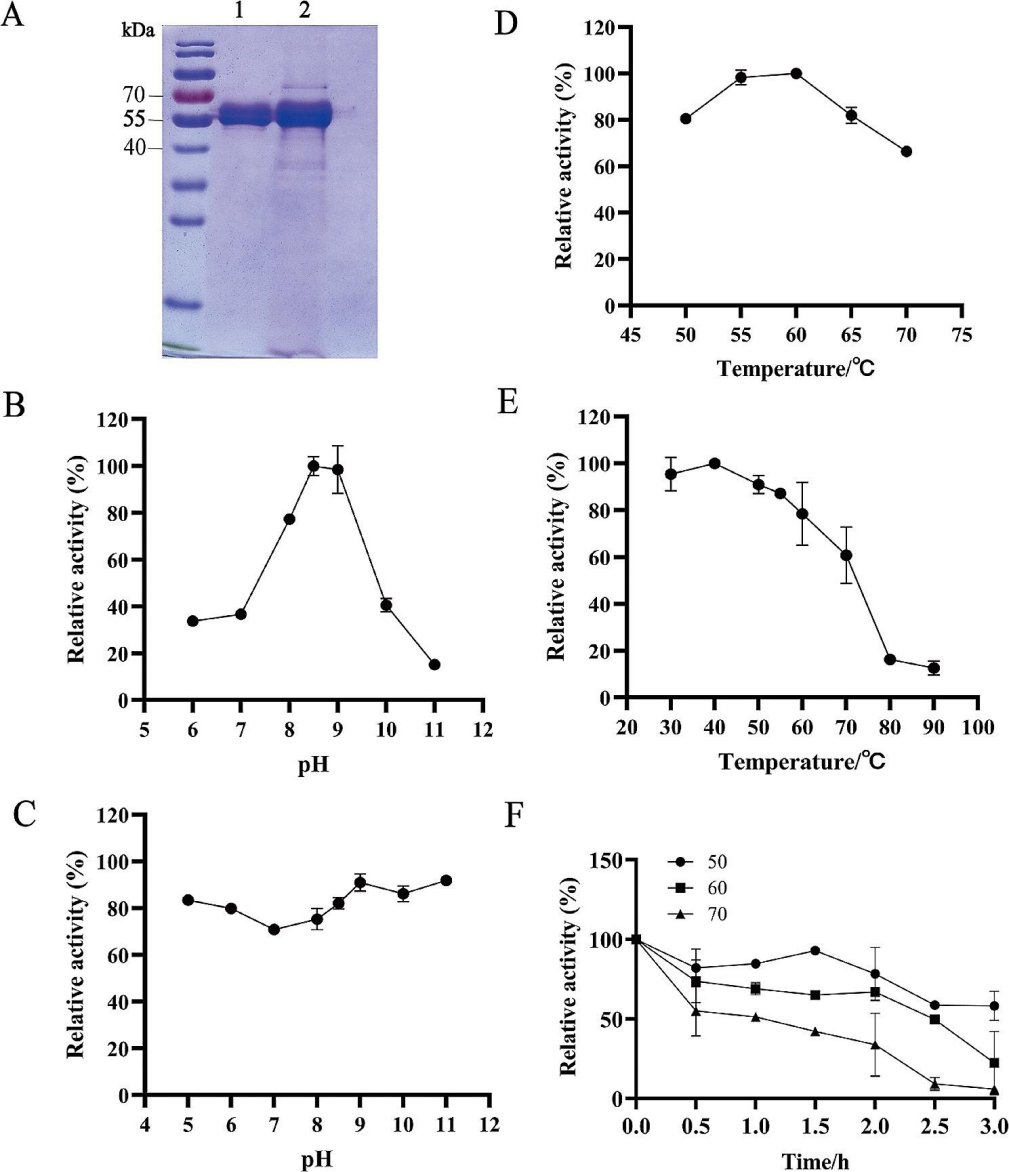
Enzymatic properties of PMGL-Ba. (**A**) SDS-PAGE analysis; 1:Purified fermentation supernatant; 2:Fermentation supernatant. (**B**) Determination of optimum pH (incubation in pH 5–11 buffer for 1 h). (**C**) pH stability (purified enzymes were incubated between pH 5.0–12.0 at 4℃ for 12 h). (**D**) Determination of optimum temperature (in a range of 50℃-70℃ in pH 8.0). (**E**) Temperature stability (Incubate in Tris-HCl buffer (pH 8.0) at 30–90℃ for 1 h). (**F**) PMGL-Ba temperature half-life

The collected fermentation supernatant was purified and analyzed for enzymatic characterization of PMGL-Ba. The purified PMGL-Ba was analyzed by SDS-PAGE, revealing an apparent molecular weight of about 55 kDa, consistent with the theoretical molecular weight of 54.6 kDa, indicating the successful heterologous secretory expression of recombinant PMGL-Ba using P. pastoris X33(Fig. 2A).

The enzyme activity of PMGL-Ba exhibited an initial increase followed by a decrease with increasing pH, pinpointing the optimal reaction pH as 8.5 (Fig. 2B). PMGL-Ba retained more than 80% of its total activity within the pH range of 8.0-9.5. However, the enzyme activity decreased rapidly beyond pH 9.0 or below 8.0. PMGL-Ba has good pH stability, maintaining 60% activity in the pH range of 5.0–11.0(Fig. 2C). The optimal temperature for PMGL-Ba was 60 °C, with relative enzyme activity exceeding 60% at temperatures between 50 and 70 °C (Fig. 2D). PMGL-Ba activity decreased with increasing temperature, and relative enzyme activity remained above 60% at temperatures below 70 °C after 1 h of exposure. As the temperature increased further, the enzyme activity gradually decreased, with only about 20% of the enzyme activity remained at the temperature of 80 ℃ (Fig. 2E). PMGL-Ba showed robust enzyme activity and stability over a broad temperature range of 30 to 80 °C, indicating its potential advantages for application in medium and high-temperature industries, such as the paper industry. When the enzyme solution was treated at 50–70 °C for 90 min, more than 50% of the enzyme activity remained. At 50 °C for 180 min, 58.26% of the enzyme activity remained. At 60 and 70 °C for 180 min, only 22% and 6% of the enzyme activity remained. The half-life of PMGL-Ba showed that PMGL-Ba displays some potential for application in the paper industry, so the main subsequent attempts were made to increase PMGL-Ba expression (Fig. 2F).

**Fig. 3:**
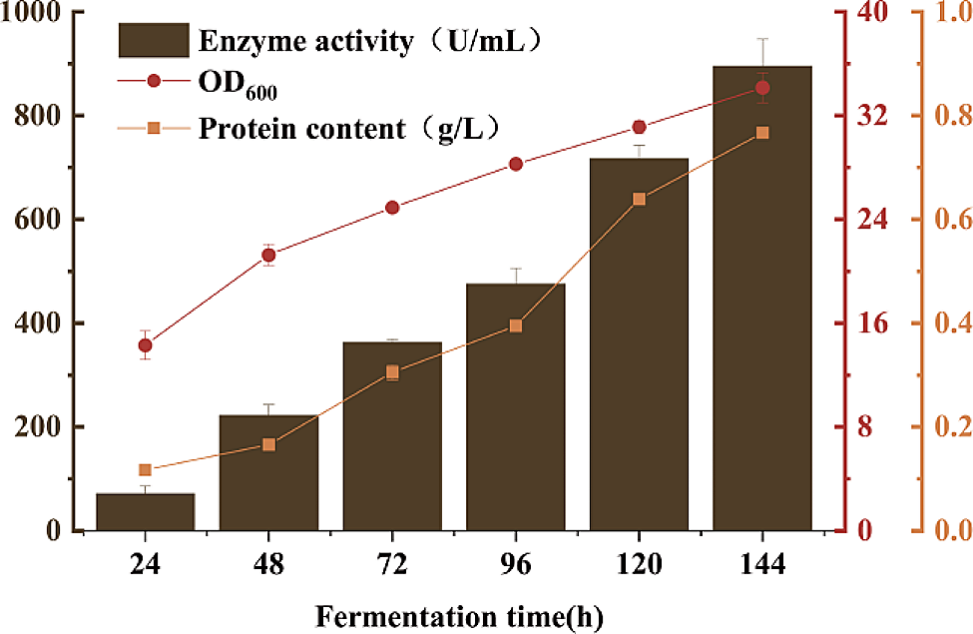
Analysis of PMGL-Ba fermentation in *P. pastoris*

The shake flask fermentation of PMGL-Ba recombinant *P. pastoris X33* was carried out, with samples taken at 24 h intervals to assess the growth of the recombinant yeast and the enzyme activity of pectin cleavage in the supernatant of the fermentation broth. With an increase in induced expression time, the OD_600_, fermentation broth supernatant enzyme activity, and protein yield of X33-PMGL-Ba gradually increased. At 144 h of induced expression time, the enzyme activity and protein concentration with esterified pectin as substrate reached 895.69 U/mL and 0.77 g/L, respectively (Fig. 3). Various strategies were followed to increase the expression of PMGL-Ba.

### Optimization of expression elements on recombinant PMGL-Ba protein production

The secretory expression of heterologous proteins in *P. pastoris* often involves the utilization of the *S. cerevisiae* α-factor mating pheromone (α-MF) from Saccharomyces cerevisiae. Existing studies have explored the impact of signaling peptides on the secretion of recombinant proteins, revealing varying efficiencies of signaling peptides for different exogenous proteins. Studies on the effect of signal peptides on exogenous protein secretion can be broadly categorized into two groups: modification of existing signal peptides and the discovery of new signal peptides. The commonly used α-MF comprises a two-stage signal, consisting of a 19-amino-acid pre region and a 66-amino-acid pre region at the N-terminal end, demonstrating effective secretion for most exogenous proteins (Barrero et al. 2018). Several studies have sought to enhance the secretion efficiency of α-MF various modifications, such as αMF-CC (αM), and α_opt_ obtained after optimization and point mutation of the αMF sequence, resulting in improved protein secretion (Ahn et al. 2016; Aza et al. 2021; Ito et al. 2022). Another approach involves utilizing more efficient sequences to replace parts of the αMF. Juan J et al. designed combined secretion signals, such as the one consisting of the Saccharomyces cerevisiae Ost1 signal sequence and the α-factor PRO region, which has demonstrated superior secretion ability compared to the α-factor signal sequence (Barrero et al. 2018). In yeast, secreted proteins are targeted to the endoplasmic reticulum (ER) by either the co-translational pathway or the “post-translational” pathway. The co translational pathway is mediated by the signal recognition particle (SRP), while the “post-translational” pathway, independent of SRP, requires the intact membrane protein Sec62p for ER targeting. However, more hydrophobic signaling sequences utilize the SRP pathway to target the ER in order to increase secretion efficiency (Walter et al. 2011). Ost1p, a type I intact membrane protein with a cleavable signal sequence, plays a role in co-translational translocation to the ER (Willer et al. 2008). Subsequent bioreactor-scale fermentation has further confirmed that specific recombinant signal peptide promotes protein secretion (Barrero et al. 2021). To avoid variability in signal peptide efficiency for different protein secretion, the discovery of new signal peptides is essential (Liang et al. 2012; Sastry et al. 2014). Systematic analysis of yeast genomes and secretomes has led to the identification of novel signal peptides with more efficient secretion than α-MF, such as the signal peptide derived from the *P. pastoris* PAS_chr3_0030 gene (PC0) and the signal peptide NCW2 in Saccharomyces cerevisiae (Shen et al. 2022; Wang et al. 2015; Zou et al. 2022).

**Fig. 4:**
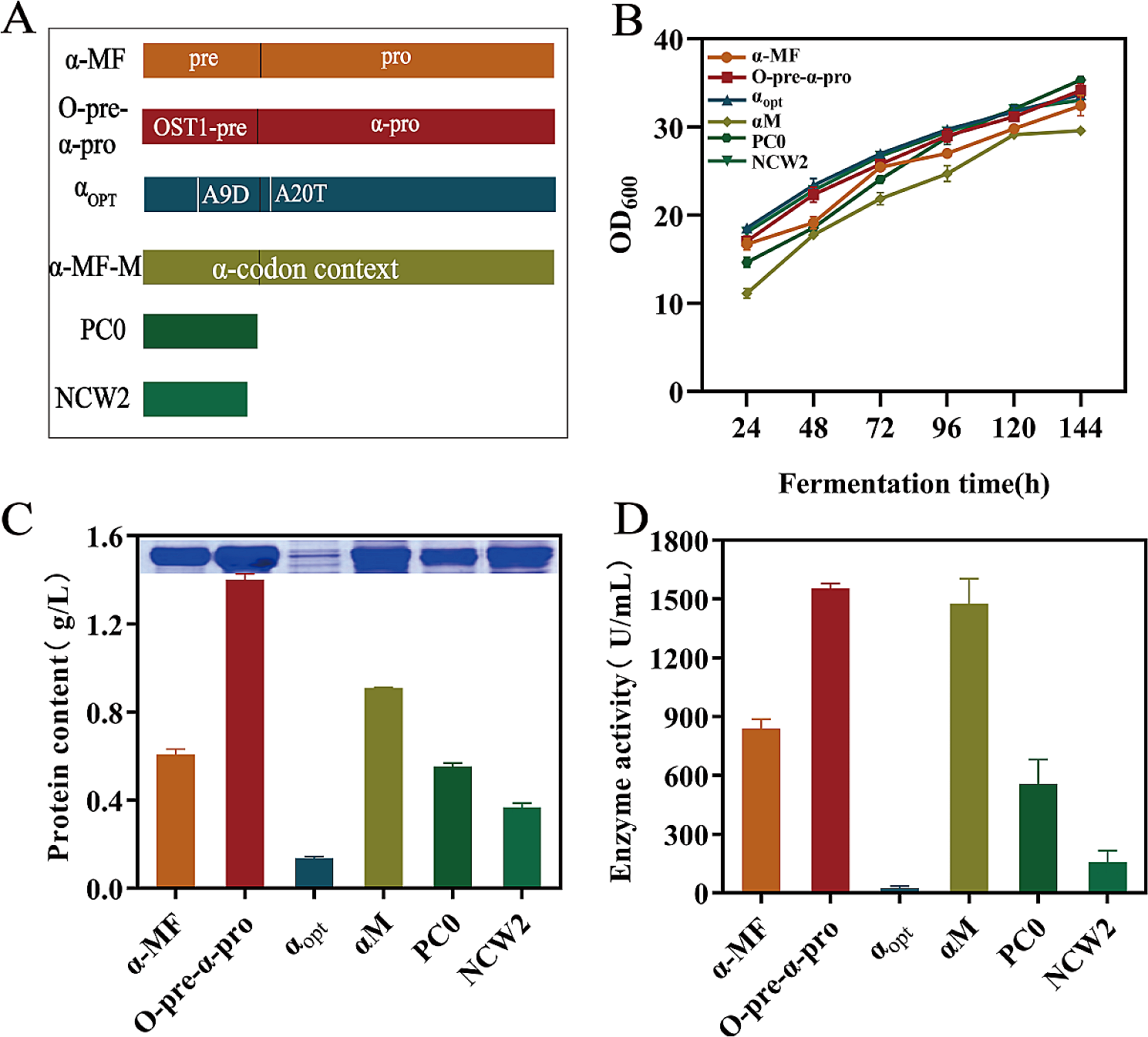
Effects of different signaling peptides on PMGL-Ba expression. (**A**) Selection of signal peptides. (**B**) Growth curves of strains corresponding to different signal peptides. (**C**) Effect of different signal peptides on total protein concentration of PMGL-Ba and SDS-PAGE analysis. (**D**) Effect of different signaling peptides on PMGL-Ba enzyme activity

In this study, six distinct types of secreted signaling peptides, namely pPIC-α-MF-PMGL-Ba, pPIC-O-pre-α-pro-PMGL-Ba, pPIC-α_opt_-PMGL-Ba, pPIC-αM-PMGL-Ba, pPIC-PC0-PMGL-Ba, and pPIC-NCW2-PMGL-Ba, were selected to investigate their effects on PMGL-Ba production. As shown in Fig. 4, results from shake flask fermentation revealed a significant enhancement in pectinase secretion with O-pre-α-pro and aM compared to α-MF. Conversely, PMGL-Ba secretion by α_opt_ and NCW2 was lower than that of α-MF. The signaling peptide PC0 exhibited secretion levels similar to α-MF. Notably, the protein concentration and enzyme activity of Ba secreted by the signal peptide O-pre-α-pro were 1.40 g/L and 1554.26 U/mL, respectively, representing a 130.71% and 98.13% increase compared to the departure strain pPICZαA-PMGL-Ba.

*Pichia pastoris* is a methylotrophic yeast, and the expression of exogenous proteins using methanol as a carbon source is tightly regulated by the methanol utilization pathway (MUT) promoter. The transcription level of exogenous protein genes is closely linked to the strength of promoter regulation(Püllmann and Weissenborn 2021). The AOX1 promoter is commonly used and is often the promoter of choice for its strong induction by methanol. However, the mechanism of action of the AOX1 promoter strictly induced by methanol is not fully understood(Berg et al. 2013; Wang et al. 2016).

To unravel the mechanism of AOX1 promoter action, previous studies have utilized mutation screening, followed by sequence analysis, or adjusted regulatory factors of other carbon sources (Hartner et al. 2008; Zhan et al. 2017). In this study, three MUT strong promoters, reported to be more effective than AOX1 in inducing exogenous proteins, were selected. The first, AOXm, involved deleting a transcription factor sequence associated with glucose repression and copy addition of a positive cis-acting element sequence after AOX1 deletion (Hartner et al. 2008; Li et al. 2015). The second, from the study by Thomas Vogl et al., utilized genes from the pentose phosphate pathway (PPP) and the defense against reactive oxygen species (ROS) in the MUT pathway to provide strong promoters that somewhat exceed AOX1, such as the promoter of the gene encoding peroxisomal membrane protein (PMP20) (Yurimoto et al. 2000; Vogl et al. 2015). The third is formaldehyde dehydrogenase 1 (FLD1), a key enzyme in metabolizing methanol as a carbon source (Nakagawa et al. 2004; Wang et al. 2019). The promoters of their encoding genes show similar yields for FLD1 and AOX1 in exogenous protein expression, with FLD1 even surpassing AOX1 in some proteins (Püllmann and Weissenborn 2021; Wang et al. 2016).

**Fig. 5:**
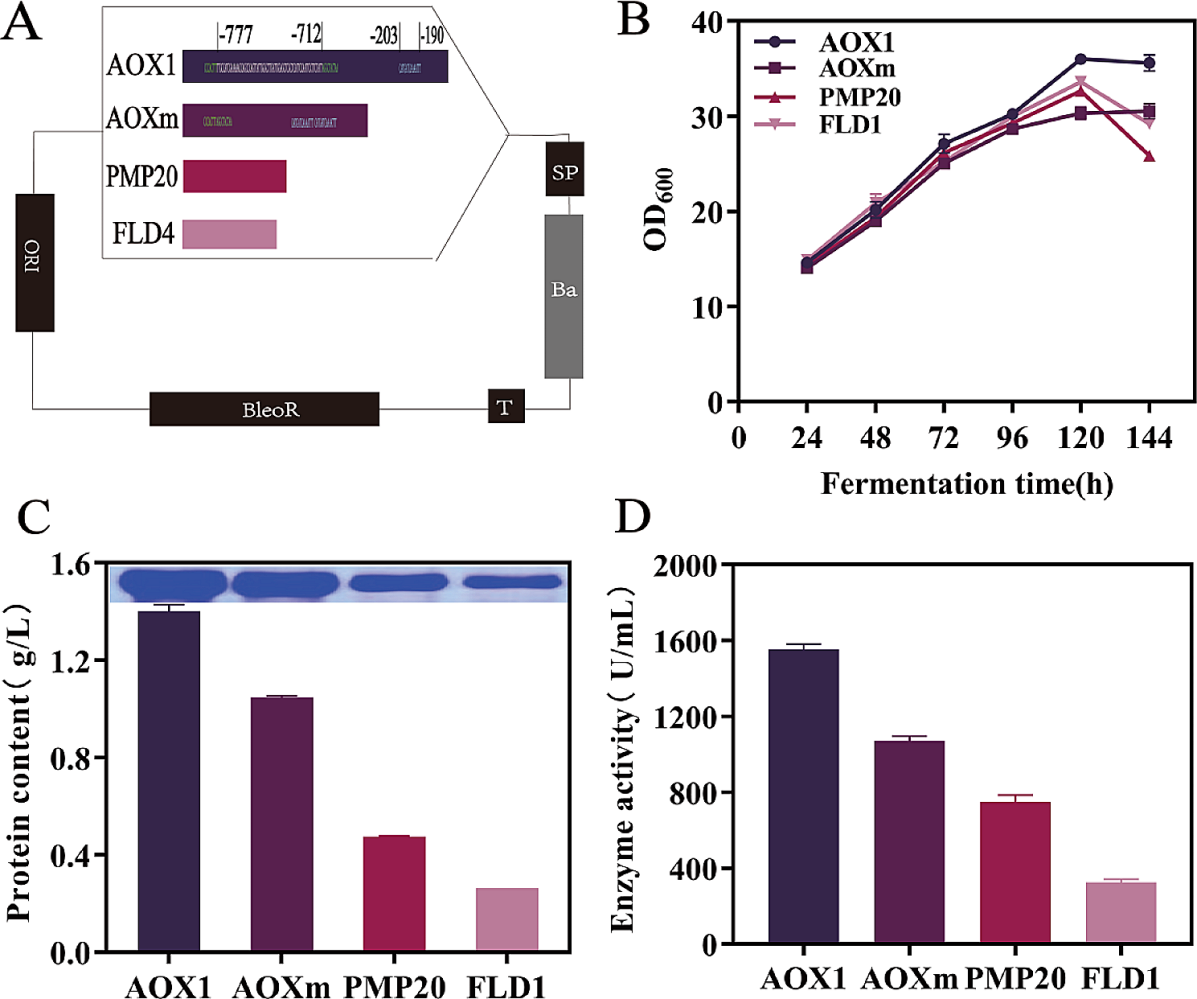
Effects of different promoters on PMGL-Ba expression. (**A**) Selection of promoters. The length of the graph represents the length of the promoter base sequence. In this case, AOXm is derived by deleting a base sequence (white) and copying a base sequence (light blue) from the AOX1 sequence. (**B**) Growth curves of strains corresponding to different promoters. (**C**) Effect of different promoters on total protein concentration of PMGL-Ba and SDS-PAGE analysis. (**D**) Effect of different promoters on PMGL-Ba enzyme activity

In this study, all four promoters mentioned above were individually employed for PMGL-Ba expression, and the most suitable promoter for PMGL-Ba gene was selected. As depicted in Fig. 5, AOX1 demonstrated obvious advantages in PMGL-Ba expression. Biomass analysis further indicated a notable decreasing trend for strains PMP20 and FLD1, suggesting that AOX1 remains the most suitable promoter for PMGL-Ba gene expression in this context.

Püllmann et al. experimentally verified that the combination of promoter and signaling peptide has some synergistic effect, rather than just considering promoter strength and signaling peptide fitness as isolated factors. Selection of a strong promoter and a suitable signal peptide for protein secretion is effective in most cases. In this study the combination of the signal peptide O-pre-α-pro and the strong promoter AOX1 was more favorable for the secretory expression of PMGL-Ba (Püllmann and Weissenborn 2021).

### Enhancemengt of gene dosage on recombinant PMGL-Ba protein production

In the expression of exogenous proteins in *P. pastoris*, constructing high-copy strains is often a straightforward and effective approach to enhance protein production. Various methods exist for constructing multicopy strains, with a common strategy involving the in vitro construction of multiple expression cassettes in tandem, subsequently integrated onto the *P. pastoris* genome. In order to obtain higher copy number strains, different screening markers can be employed for sequential integration into the *P. pastoris* genome (Wang et al. 2021; Yang et al. 2016). Another prevalent method involves the gradual increase of antibiotic concentration, with commonly used antibiotics such as zeocin and antibiotic G418; the screening concentration is usually higher than 100 µg/mL, with some reaching up to 2000 µg/mL (Kuo et al. 2015; Sha et al. 2013; Song et al. 2019). However, zeocin is a relatively expensive antibiotic, and its screening process is intricate and time-consuming. Another approach involves the Cre/lox recombination system, using a screening marker for multiple integration, but this method suffers from a prolonged cycle time (Li et al. 2017). In addition, Xia et al. constructed a novel double plasmid system using a combined strategy of genomic integration and heterologous expression (Xia et al. 2021).

**Fig. 6:**
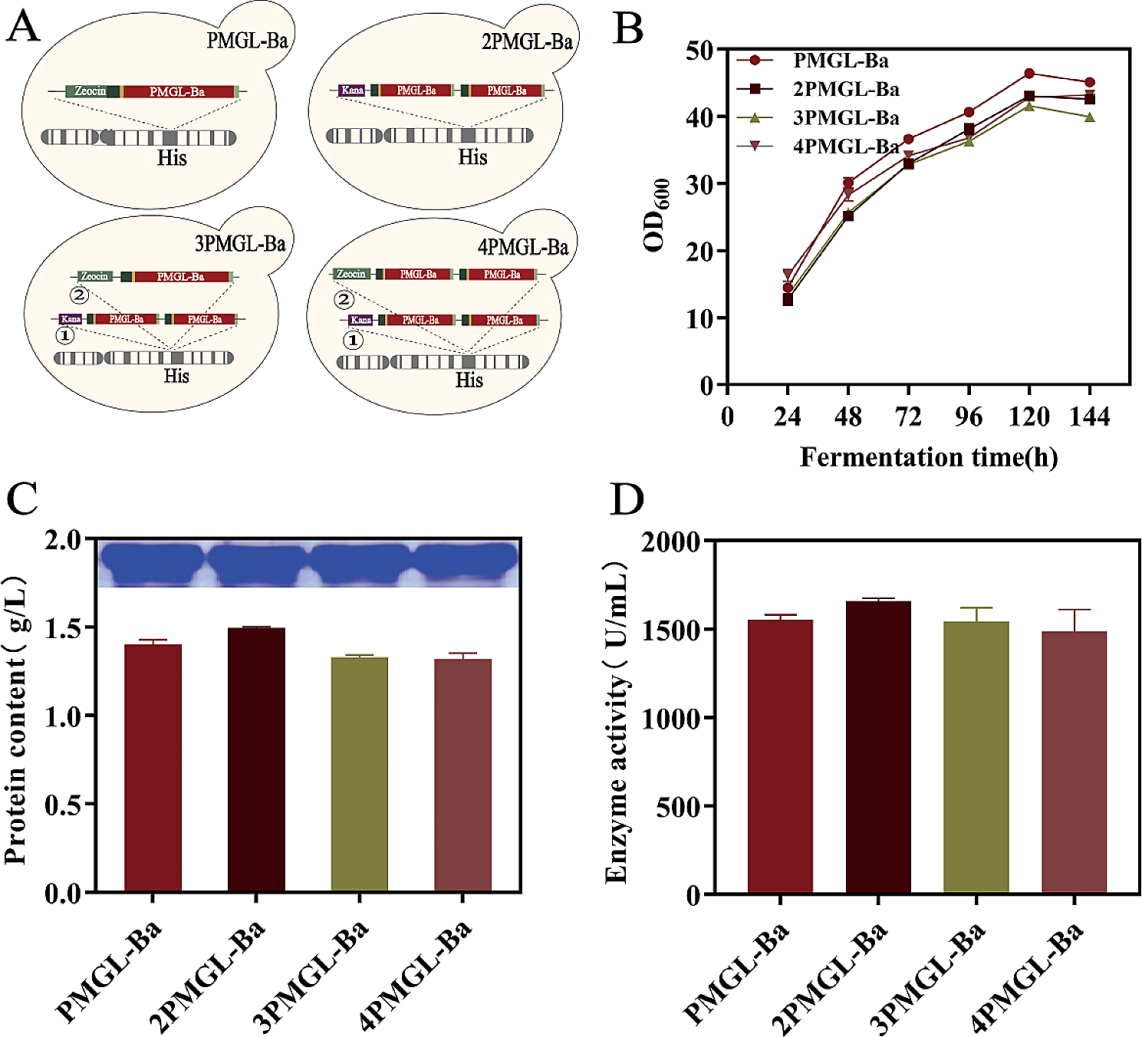
Effects of gene dosage on PMGL-Ba expression. (**A**) Schematic of different gene doses. Red is the PMGL-Ba gene, light green and purple are screening markers, and ①② is the gene integration order. (**B**) Growth curves of strains corresponding to different gene doses. (**C**) Effect of different gene doses on total protein concentration of PMGL-Ba and SDS-PAGE analysis. (**D**) Effect of different gene doses on PMGL-Ba enzyme activity

In this study, homologous recombination was employed to construct three strains with different screening markers, namely GS115-2PMGL-Ba, GS115-3PMGL-Ba, and GS115-4PMGL-Ba, which were sequentially integrated onto the Picot yeast genome using different screening markers. This method effectively reduced the time required for constructing multiple copies by 2–3 days, streamlining the identification process post-integration into the yeast genome. As illustrated in Fig. 6, GS115-2PMGL-Ba exhibited improvements over GS115-PMGL-Ba, showcasing increased protein concentration and enzyme activity. However, GS115-3PMGL-Ba demonstrated a significant decrease in yield compared to GS115-2PMGL-Ba. In shake flask fermentation, the protein concentration and enzyme activity of GS115-2PMGL-Ba reached 1.50 g/L and 1658.01 U/mL, respectively, representing a 6.7% and 6.69% increase compared to X33-PMGL-Ba.

### Overexpression of cofactors on recombinant PMGL-Ba protein production

In the realm of protein expression pathways, alterations in chaperone protein levels can exert influence across multiple stages, encompassing transcription, translation, folding, and secretion. However, the impacts of such alterations are often protein-specific, and determining the singularly most crucial chaperone or the optimal combination remains ambiguous (Samuel et al. 2013). In this study, we expanded on GS115-2Ba by overexpressing 11 cofactors previously investigated for their roles in promoting protein expression.

A pivotal step in exogenous protein expression in *P. pastoris* is the translation process, where translation initiation serves as the rate-limiting step in the translation process. Various translation initiation-associated factors have been targeted to study the impact of translation efficiency. Notably, Jennifer Staudacher et al. demonstrated that overexpression of certain translation factors, particularly Pab1 and eIF4G, significantly enhances exogenous protein production in *P. pastoris*. The synergistic effect was more pronounced when all four loop-closing related factors, eIF4E, eIF4A, eIF4G and Pab1, were simultaneously overexpressed (Staudacher et al. 2022). In a previous study in our laboratory, Zheng et al. also proposed a novel yeast transcription factor, tentatively named Fhl1p, implicated in the regulation of rRNA processing genes and genes related to ribosomal small/large subunit biogenesis genes. This factor was found to facilitate the translation efficiency of exogenous proteins (Zheng et al. 2019).

Correct protein folding is a critical step in *P. pastoris*, particularly during post-translational translocation in the endoplasmic reticulum (ER). Newly synthesized peptides entering the ER must maintain an unfolded or loosely folded form after release from the ribosome, preventing aggregation before translocation. This process involves binding to cytoplasmic chaperones such as Ssa1 and Ydj1. In contrast, the ATPase activity of the ER luminal chaperone Kar2 powers post-translational translocations, promoting protein folding as well as targeting misfolded proteins to ERAD (Delic et al. 2013).

In response to endoplasmic reticulum stress induced by the overexpression of exogenous proteins, especially in multicopy strains, a common approach to alleviate these stress effects is the overexpression of cofactors. The HSP30 gene has been shown to play a crucial role in mitigating the repression of target gene transcript levels induced by high-intensity promoters. Besides, HSP30 gene expression is simultaneously regulated by the transcription factors HSF1, MSN2, and MSN4. Notably, the transcription factor HSF1 positively affects alleviating unfolded protein stress (Cui et al. 2023). Overexpression of the transcription factor Hac1p is frequently selected during chaperonin assisted secretory protein folding. In multicopy strains, the overexpression of exogenous proteins induces cellular stress responses, such as the common unfolded protein response (UPR), activated by unconventional splicing of HAC1 mRNA in yeast. The spliced HAC1 mRNA encodes an active transcription factor, Hac1p, which binds to UPR response elements in the promoters of UPR target genes, and it induces the expression of other chaperone proteins that assist in protein folding and protein transport (Liu et al. 2020; Guerfal et al. 2010).

**Fig. 7:**
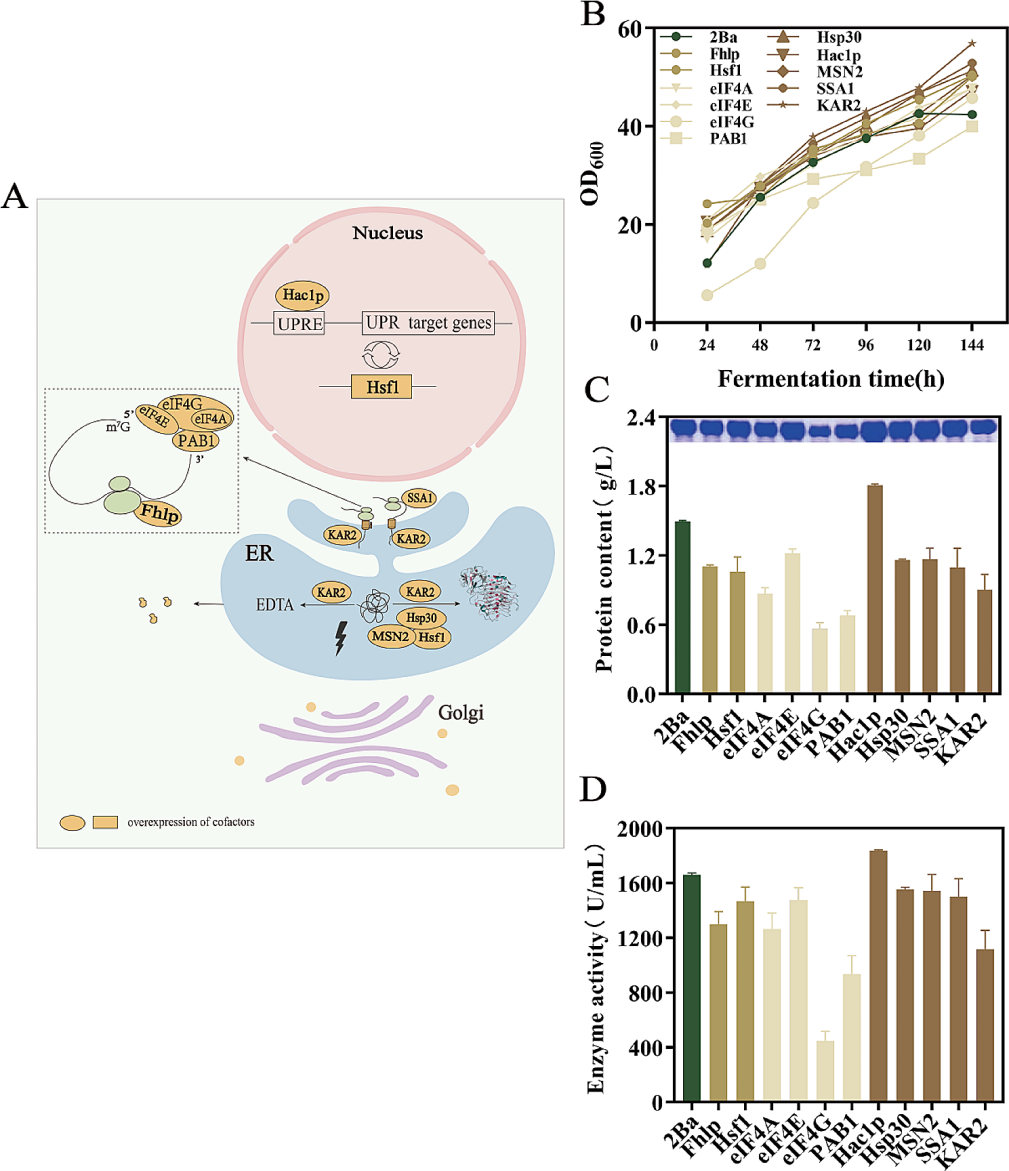
Position of different protein cofactors in the protein secretory expression pathway. (**A**) Schematic of different gene doses. (**B**) Growth curves of strains corresponding to overexpression of different protein cofactors. (**C**) Effect of overexpression of different protein cofactors on total protein concentration of PMGL-Ba and SDS-PAGE analysis. (**D**) Effect of overexpression of different protein cofactors on PMGL-Ba enzyme activity

In this study, we systematically overexpressed the 11 cofactors previously discussed, building upon the foundation of GS115-2PMGL-Ba. Interestingly, the Fig. 7 indicates that only overexpression of Hac1p increased the protein expression, while overexpression of the other ten cofactors decreased the protein expression. In shake flask fermentation, the protein concentration and enzyme activity of strain GS115-2PMGL-Ba: Hac1p were 1.81 g/L and 1835.53 U/mL, respectively. Compared with GS115-2PMGL-Ba, the protein concentration and enzyme activity increased by 20.85% and 10.7%.

In previous studies we also observed different molecular chaperones that play a positive role. In the study of Liu et al. co-expression of most molecular chaperones showed a decrease in protein expression, similar to the results of this paper (Liu et al. 2023). In contrast, co-expression of most molecular chaperones had a positive effect on protein secretion in Wang et al. (Wang et al. 2023b). When using co-expression of molecular chaperones as a strategy to increase protein expression, attempts should be made to select more molecular chaperones to study the effect on protein expression. Also mining more molecular chaperones related to protein secretion is a long-term research topic.

### High density fermentation in 5 L bioreactors

After identifying the highest PMGL-Ba yielding strain, GS11-2PMGL-Ba-Hac1p, through the different strategies mentioned above, it was activated and cultured into a seed solution. This seed solution was then introduced into a 5 L fermenter containing 2 L of BSM basal medium to initiate the batch culture phase. The glycerol feed-batch phase commenced after a dissolved oxygen rebound at 28 h. Glycerol replenishment was stopped when the yeast density reached OD_600_ = about 300, indicating complete glycerol consumption. Subsequently, the methanol feed-batch phase commenced, with samples taken every 8 h, continuing until the completion of 72 h.

**Fig. 8:**
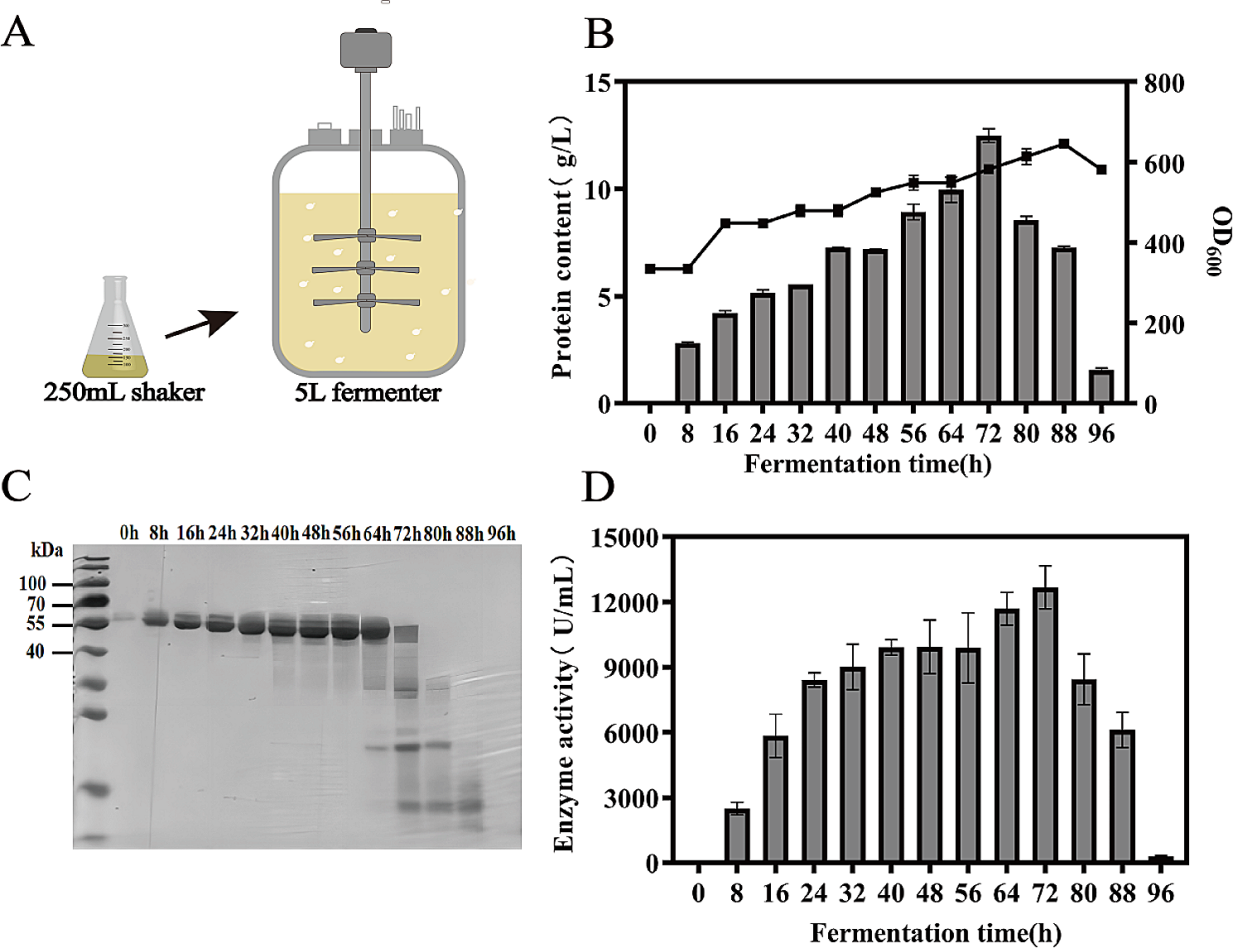
PMGL-Ba high density fermentation. (**A**) 5 L type fermenter high density fermentation. (**B**) Changes in biomass and total protein concentration over time in high-density fermentations. (**C**) SDS-PAGE analysis of supernatants after 20-fold dilution at different times in high-density fermentation. (**D**) Changes in PMGL-Ba activity at different times in high-density fermentation

As shown in the Fig. 8, the high-density fermentation of GS115-2PMGL-Ba-Hac1p in a 5 L fermenter for 72 h resulted in a substantial increase in protein concentration to 12.49 g/L, representing a remarkable 591.51% improvement compared to shake flask fermentation. Simultaneously, enzyme activity increased to 12668.12 U/mL, reflecting a 595.53% increase compared to shake flask fermentation. Previous experimental findings indicated protein degradation in the late stage of fermentation. To expedite the fermentation process, we increased the starting cell density of the induction stage of methanol flow addition from OD_600_ = 200 to OD_600_ = 300, but protein degradation is still significant.

Degradation of exogenous proteins is a serious problem in high-density fermentation of *P. pastoris*. Zhang et al. described the problem of protein degradation and gave corresponding solutions from the cultivation level, the cell level and the protein level. The degradation of exogenous proteins is closely related to the protein hydrolases secreted by *P. pastoris*, and three types of protein hydrolases are generally present in *P. pastoris*: the cytosolic proteasome, vacuolar proteases, and proteases located in the secretory pathway. The degradation of exogenous proteins by proteolytic enzymes is mainly prevented by inhibition of protease activity, reduction of protease expression and non-recognition by proteases (Zhang et al. 2007).

At the cultivation level, the main focus is on reducing protease activity by controlling pH, temperature, nitrogen source and the addition of proteolytic enzyme inhibitors. In the study by Sun et al., the effect of degradation of recombinant human serum albumin produced by the fermentation of *P. pastoris* was investigated by controlling temperature, pH and the addition of nitrogen source. The results showed that pH and nitrogen source significantly improved the degradation of albumin, while temperature had little effect (Sun and Chen 2004). Furthermore, the addition of protein hydrolase inhibitors on an industrial scale is associated with higher costs compared to other options (Zhang et al. 2007).

At the cellular level, knocking out major proteolytic enzymes is the most important strategy for reducing degradation. Currently, the disruption of genes encoding proteinase A (PEP4), proteinase B (PRB1) and yapsin 1 (YPS1) is relatively widespread and has a significant positive effect on the degradation of exogenous proteins recombinantly introduced into *P. pastoris* (Marsalek et al. 2017; Wang et al. 2023b; Wu et al. 2013; Yao et al. 2009). Finally, at the protein level, this occurs mainly through the modification of amino acid sites recognized by protein hydrolases (Zhang et al. 2007).

In the high-density fermentation process described in this paper, attention was paid to low-temperature induction and increasing the induction OD_600_ to avoid degradation of recombinant proteins, but no improvement was obtained. We also knocked out the PEP4 and PRB1 genes in the highest-yielding strains, and the results in the shake flask showed that knocking out the two genes did not significantly improve protein degradation and the production of PMGL-Ba decreased significantly (data not shown).

Protein degradation can be attenuated by pH control in subsequent studies. Our tentative solution is to avoid degradation by shortening the fermentation cycle and aborting the fermentation in time, which also balances cost and yield to some extent. It is also necessary to analyze more protein hydrolytic enzymes related to protein degradation during high-density fermentation.

## Methods

### Strains, media, and reagents

The molecular kits used in this experiment were purchased from Magen Biotechnology Co.,Ltd. Restriction endonucleases were purchased from Thermo Fisher Scientific (Shanghai,China). The homologous recombinant enzyme was purchased from Beijing Jinsha Biotechnology Co. Chemical reagents were purchased from Tianjin Damao Chemical Reagent Factory. Pectin was purchased from McLean. Recombinant plasmids and recombinant strains were constructed using *Escherichia coli Top10* and *P.pastoris* as hosts. The medium media and their compositions used in this experiment were LB (0.5% yeast extract, 1% peptone and 1% NaCl), LBL (0.5% yeast extract,1% peptone and 0.5% NaCl), YPD (1% yeast extract, 2% peptone and 2% glucose), MD (1.34% YNB and 2% glucose), BMGY (1% yeast extract, 2%.

peptone, 1.34% YNB, 0.1 mM Potassium phosphate buffer pH 6.0 and 1% glycerol) and BMMY (1% yeast extract, 2% peptone, 1.34% YNB, 0.1 mM Potassium phosphate buffer pH 6.0 and 1% methanol).

### Gene mining for putative xylanase and sequence analysis for target gene

Gene mining for PMGL was performed with the help of enzyme resource mining software EnzymeMiner (Damborsky et al. 2020). Two high enzyme activity pectin lyases Q01172 and P94449 of medium alkaline and medium high temperature were used as probe sequences. Additionally, the protein sequences of three PMGLs, Q2TXS4, AY825251, and R9XVW6, were used as auxiliary references for sequence search. The initial set of sequences of potential PMGLs was obtained by performing iterative site-specific homology searches in the NCBI database. The key residues of PMGL were then used as filtering criteria to filter sequences with mismatched key residues in the homology search results. The final selection was based on the sequence similarity and sequence characterization provided by EnzymeMiner, the sequence similarity to the probe template (with a threshold value of 30-70%), the predicted degree of soluble expression of the protein, and the type of microorganism from which the protein was derived. A phylogenetic tree was constructed between the selected sequences and the probe sequences by neighbor-joining method using MEGA-X software to analyze the homology of the sequences. The protein structure of PMGL-Ba was modeled by SwissModel homology. Sequence comparison analysis was performed using Jalview. The basic physicochemical properties of *Bacillus licheniformis* pectin lyase were predicted using ProtParam.

### Heterologous expression of PMGL-Ba in *P. pastoris*

The PMGL gene (NCBI: WP_009329358.1) derived from *Bacillus licheniformis* was mined by homologous sequence comparison. Subsequently, the PMGL gene was codon optimized and constructed in the pPICZαA vector, named pPICZαA-PMGL-Ba. The recombinant plasmid was then transformed into *E. coli Top10* receptor cells, coated to solid medium containing zeocin antibiotic in LBL and cultured for 12–14 h. Single colonies, correctly identified by PCR, were transferred to liquid medium of LBL and cultured for 12–14 h. Plasmids were extracted and sequenced according to the kit. The plasmids with correct sequencing were linearized and transformed into *P. pastoris* by electroporation and selected on YPDZ plates, and incubated at 30 °C for 3 d. Yeast single colonies with correct colony morphology were identified by PCR.

The recombinant *P. pastoris* strains were transferred to 10 mL of BMGY medium at 30℃ for 20–24 h, and then transferred to 25 mL of BMMY medium according to the starting OD_600_ as 1. Samples were taken every 24 h and 1% (v/v) methanol was added. After 144 h of fermentation, the samples were transferred to 50mL centrifuge tubes. After centrifugation, the supernatant was taken for subsequent experiments.

The collected fermentation supernatant was centrifuged at high speed (10,000 rpm, 4℃, 10 min), and then passed through a 0.22 μm ultrafiltration membrane. Gradient purification of fermentation broth supernatant using Ni-NTA column chromatography, and the eluate was collected. The protein content of the fermentation supernatant and purified PMGL-Ba were determined using the Bradford method, with bovine serum albumin (BSA) as the standard. Finally, the supernatant was diluted 2-fold and 20 µL was taken for SDS-PAGE analysis.

### Enzymatic properties of PMGL-Ba

In this study, we mainly referred to the activity assays for pectin lyase reported and the DNS method described in the literature and comprehensively selected the assay suitable for the pectin lyase enzyme activity in this study (Chen et al. 2022, 2023; Zhang et al. 2022). Initially, 0.5% pectin was accurately weighed and dissolved in Tris-HCl buffer (50 mM, pH 8.0). The fermentation supernatant was appropriately diluted. Subsequently, 190 µL of the pectin solution was placed in a thermostatic reactor to preheat for 10 min, and 10 µL of sample was added to react for 10 min. 300 µL of DNS solution was added, boiled for 5 min and then cooled down immediately, and 200 µL of the reaction solution was taken to measure the absorbance value at 540 nm. One unit (U) of enzyme activity was defined as the amount of pectin lyase releasing 1 µmol of reducing sugar per minute under the conditions described above.

The optimal pH value of PMGL-Ba was measured by determining the activity between pH 5.0–11.0 in 60 °C. To determine pH stability of PMGL-Ba, purified enzymes were firstly incubated between pH 5.0–12.0 at 4 °C for 12 h. Next, residual activity of PMGL-Ba was further measured using above method. Buffers used were 100 mmol/L PBS buffer (pH 6.0), 50 mmol/L Tris-HCl buffer (pH 7.0–9.0) and Glycine-sodium hydroxide buffer(pH10-11). Optimal temperature of PMGL-Ba was investigated by measuring the activity in a range of 50–70 °C in pH 8.0. To determine the thermostability of PMGL-Ba, enzymes were pre-incubated at 30–90 °C for 1 h pH 8.0.

### Construction of highly expressed PMGL-Ba recombinant strains

To investigate the expression of PMGL-Ba by secreted signaling peptides, five signaling peptides were selected based on literature research as OST1-pre-αMF-pro(O-pre-α-pro), α_opt_, αM, PAS_chr3_0030 gene (PC0) and NCW2, respectively. Subsequently, α-MFs were sequentially replaced by homologous recombination, resulting in constructs named pPIC-O-pre-α-pro-PMGL-Ba, pPIC-αopt-PMGL-Ba, pPIC-αM-PMGL-Ba, pPIC-PC0-PMGL-Ba, and pPIC-NCW2-PMGL-Ba. In parallel, three strong promoters, AOXm, FLD1, and PMP20, were also substituted for AOX1 by homologous recombination to explore the expression of the promoters on the proteins, named pPIC-AOXm-PMGL-Ba, pPIC-FLD1-PMGL-Ba, and pPIC-PMP20-PMGL-Ba, respectively. To facilitate in vitro construction of multiple copies, the PMGL-Ba gene expression cassette was constructed on the PHK-PMGL-Ba and pPIC-Cre/lox-PMGL-Ba plasmid vectors. PHK-2PMGL-Ba and pPIC-Cre/lox-2PMGL-Ba were constructed using homologous recombination. Three-copy and four-copy recombinant strains were constructed by screening markers differentially integrated sequentially on the yeast genome. To further promote protein expression, eleven cofactors were overexpressed in multicopy strains as Fhlp, Hsf1, eIF4A, eIF4E, eIF4G, PAB1, Hsp30, Hac1p, MSN2, SSA1, and KAR2, and were named as PHK-2PMGL-Ba-Fhlp, PHK-2PMGL-Ba-Hsf1, PHK-2PMGL-Ba-eIF4A, PHK-2PMGL-Ba-eIF4E, PHK-2PMGL-Ba-eIF4G, PHK-2PMGL-Ba-PAB1, PHK-2PMGL-Ba-Hsp30, PHK-2PMGL-Ba-Hac1p, PHK-2PMGL-Ba-MSN2, PHK-2PMGL-Ba-SSA1, and PHK-2PMGL-Ba-KAR2, respectively. The plasmids, confirmed by correct sequencing, were linearized and transformed into *P. pastoris* by electroporation and selected on MD plates or YPDZ plates, and incubated at 30 °C for 3 d. Yeast single colonies with correct colony morphology were identified by PCR. The recombinant *P. pastoris* strains were fermented as described above.

### Fed-batch fermentation in a 5 L fermenter

The screened high yielding strains were first streaked from the preservation tubes to YPD solid medium for 3 d activation. A single colony was picked into 10mL YPD liquid medium and incubated in shaker at 30℃ for 20–24 h to form a primary seed solution. Then the second seed liquid was obtained by transferring to 160mL YPD medium at 4% inoculum volume and culturing in shaker at 30℃ for about 20 h. The resulting seed solution was transferred to a 5 L fermenter containing 2 L of BSM medium, and 8% inoculum was introduced along with 8.7 mL of aseptically added PTM_1_ (micronutrient salt). Temperature was controlled at 30 °C, and pH was set at 5.5. The glycerol feed-batch phase commenced upon depletion of glycerol in the glycerol batch phase, accompanied by a rebound in dissolved oxygen. Glycerol addition ceased when OD_600_ = 300, signaling the transition to the methanol fed-batch phase. During this phase, temperature was 25 °C, pH = 6.0, and the induction time was 72 h. Cell biomass, protein concentration, and enzyme activity were determined by taking 10 mL samples every 8 h. At the end of fermentation, the broth underwent centrifugation, and the resulting supernatant was utilized for subsequent experiments.

## Conclusion

In this study, we elucidated the amino acid sequence of PMGL-Ba, a novel enzyme derived from *Bacillus licheniformis*, previously unreported. This was achieved through homologous sequence comparison and the identification of key amino acids using the EnzymeMiner software. For heterologous expression in *P. pastoris*, we performed codon optimization on PMGL-Ba. The enzyme exhibited an optimum pH of 8.5 and an optimum temperature of 60 ℃, displaying excellent pH and temperature stability. To enhance PMGL-Ba expression in *P. pastoris*, we employed a multifaceted approach, encompassing the selection of gene expression components, increased gene dosage, and co-expression of cofactors. Ultimately, a two-copy expression cassette, consisting of AOX1 promoter, OST1-pre-α-MF-pro signal peptide, and AOX1 terminator elements, was selected. This cassette, co-expressed with the transcription factor Hac1p, was utilized for high-density fermentation in a 5 L fermenter for 72 h. The results were impressive, with the total protein concentration reaching 12.49 g/L and the enzyme activity reaching12668.12 U/mL. This represents a remarkable 21-fold increase in total protein expression and a 15-fold increase in enzyme activity compared to the initial bacterial expression. This achievement marks the highest reported expression level of PMGL-Ba to date, providing a yeast strain with substantial PMGL-Ba production potential for future industrial enzyme applications.

### Electronic supplementary material

Below is the link to the electronic supplementary material.

Supplementary Material 1

## Data Availability

All data generated or analysed during this study are included in this published article.
